# From Diagnosis to Dialysis: Managing Primary Membranous Nephropathy in a Patient Living With Human Immunodeficiency Virus (HIV)

**DOI:** 10.7759/cureus.68036

**Published:** 2024-08-28

**Authors:** Kwasi Asamoah Opare-Addo, Stanley E Atencah, Samuel K Dadzie, Alfred Solomon

**Affiliations:** 1 Internal Medicine, Piedmont Athens Regional Medical Center, Athens, USA; 2 Nephrology, Athens Kidney Center, Oconee Medical Group, Athens, USA

**Keywords:** rituximab, interstitial fibrosis and tubular atrophy, hiv/aids, immunosuppression, primary membranous nephropathy

## Abstract

Membranous nephropathy (MN) is a significant cause of nephrotic syndrome in adults, with both primary and secondary etiologies contributing to its pathogenesis. This case report explores the clinical course of a 69-year-old African American man with human immunodeficiency virus (HIV) who developed primary MN, progressing to end-stage renal disease (ESRD) despite treatment efforts. Initially diagnosed with IgA nephropathy and HIV-associated immune complex kidney disease (HIVICK), the patient later developed anti-phospholipase A2 receptor (anti-PLA2R) antibody-positive MN. Despite immunosuppressive therapy and partial remission with rituximab, non-adherence to treatment led to disease exacerbation and eventual hospitalization for acute heart failure and worsening renal function. A subsequent renal biopsy revealed severe interstitial fibrosis and tubular atrophy, limiting further therapeutic options. This case underscores the challenges in managing MN, particularly in high-risk patients with comorbidities such as HIV, and highlights the importance of adherence to treatment and tailored management strategies to optimize outcomes in this complex condition.

## Introduction

Membranous nephropathy (MN) is the leading cause of nephrotic syndrome in non-diabetic adults, with an approximate global incidence of 8-10 individuals per million [1]. Up to 30% of all biopsy-diagnosed cases of MN are attributable to secondary causes such as autoimmune diseases, infections, medications, or malignancies [2]. The remaining cases are idiopathic. Between 50% and 80% of primary MN cases involve anti-phospholipase A2 receptor (anti-PLA2R) antibodies, while approximately 3-5% are associated with anti-thrombospondin type 1 domain containing 7A (anti-THSD7A) antibodies [3]. Patients living with human immunodeficiency virus (HIV) infection are at risk of a diverse range of kidney disorders in addition to those that affect the general population, including HIV-associated immune complex kidney disease (HIVICK) and HIV-associated nephropathy (HIVAN). Secondary MN accounts for 3-30% of HIVICK cases [4].

Longitudinal studies reveal that one-third of patients diagnosed with primary MN will advance to end-stage renal disease (ESRD) within 5-15 years [5]. Of particular clinical concern is the propensity of MN to recur post-transplantation [6]. It is therefore crucial to adopt an aggressive approach in treating MN, especially if patients have high-risk factors. This case report examines the clinical trajectory of a patient with primary MN who ultimately progressed to ESRD, aiming to elucidate the importance of adherence to medication.

## Case presentation

A 69-year-old African American man diagnosed with HIV 16 years ago who has been adherent to highly active antiretroviral therapy (HAART) (abacavir, dolutegravir, and lamivudine) with a CD4 count of 569 cells/μL and suppressed viral load presented with a two-week history of progressive lower extremity swelling extending to the groin, associated with shortness of breath and orthopnea.

**Figure 1:**
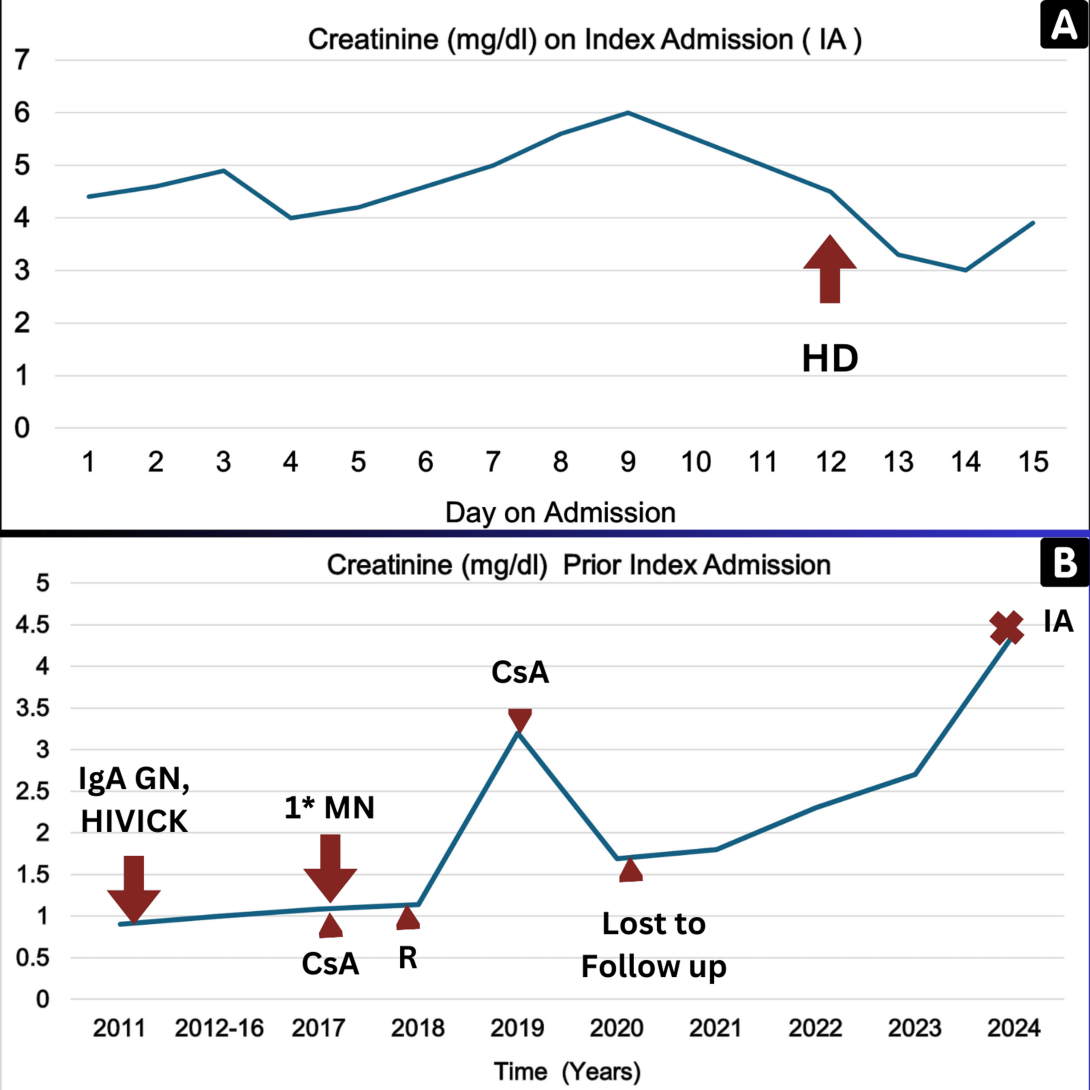
Line graphs of serum creatinine (mg/dL) over (A) the course of IA and (B) 13 years prior to IA CsA: cyclosporine; HD: hemodialysis; HIVICK: HIV-associated immune complex kidney disease; IgA GN: IgA glomerulonephritis; 1*MN: primary membranous nephropathy; R: rituximab; IA: index admission

Sixteen years ago, the patient was diagnosed with IgA nephropathy and glomerular crescent attributed to HIVICK on biopsy when he presented with hematuria. Proteinuria was persistently less than 500 mg/dL. Six years prior to index admission, the patient was also diagnosed with anti-PLA2R antibody-positive primary MN. Biopsy showed global glomerulosclerosis with minimal interstitial scarring (Figure 1). Twenty-four-hour urine protein on two occasions was 5.6 and 9.6 g. Due to his high risk of progression to ESRD including age, gender, serum albumin of 2.5 g/dL, elevated creatinine of 2.6 mg/dL, eGFR of 25 mL/min/1.73 m², and persistent significantly elevated proteinuria, the patient was placed on immunosuppression. He received cyclosporine and prednisone for six months without achieving remission. Due to concerns about increased net immunosuppression and the high risk of developing opportunistic infections, cyclosporine and prednisone were discontinued by his infectious disease doctors. The patient received two doses of rituximab, achieving only partial remission and improvement of urine protein creatinine ratio (UPCR) to 2 g/g. Due to worsening proteinuria and renal function, he was placed back on cyclosporine and prednisone. The patient was, however, lost to follow-up for close to four years and was non-adherent to immunosuppression during this period.

Three weeks prior to this index admission, the patient reestablished care at the renal clinic and was noted to have mild lower extremity swelling. Creatinine had worsened to 4.63 mg/dL from baseline after sequentially increasing lisinopril from 20 mg to 40 mg and adding dapagliflozin for persistent significant proteinuria (UPCR of 8 g/g). On admission, examination findings were pertinent for significant hypervolemia with severe lower extremity, scrotal, and sacral edema. The jugular vein was distended, and chest examination revealed bibasal crackles. A chest X-ray confirmed a moderate left pleural effusion. Creatinine was 4.35 mg/dL (Table 1 and Figure 1). An echocardiogram showed concentric hypertrophy of the left ventricle with an ejection fraction of 50-55% and grade 2 diastolic dysfunction. Renal ultrasound showed echogenic kidneys without hydronephrosis. The hepatitis viral panel was negative. Antinuclear antibodies (ANA) and antineutrophil cytoplasmic antibodies (ANCA) were negative, and the free kappa/lambda ratio was 1.05.

**Table 1:** Basic metabolic panel results over the course of admission DOA: day of admission; Na: sodium; K: potassium; Cr: creatinine; BUN: blood urea nitrogen; CO_2_: bicarbonate

Lab (units)	Results														
DOA	1	2	3	4	5	6	7	8	9	10	11	12	13	14	15
Na (135-145) (mmol/l)	144	145	143	144	142	142	142	142	141	142	144	-	-	-	-
K (3.3-5) (mmol/l)	4.1	3.8	3.8	3.6	3.3	3.7	3.7	3.4	3.1	3.4	3.7	-	-	-	-
Cr (0.57-1.00) (mg/dl)	4.4	4.6	4.9	4.0	4.2	4.6	5.0	5.6	6.0	5.5	5.0	4.5	3.3	3.0	3.9
BUN (6-24) (mg/dl)	34	36	36	33	30	32	33	33	33	34	32	-	-	-	-
CO_2 _(20-29) (mmol/l)	20	22	25	27	24	27	28	27	25	26	27	-	-	-	-

**Figure 2:**
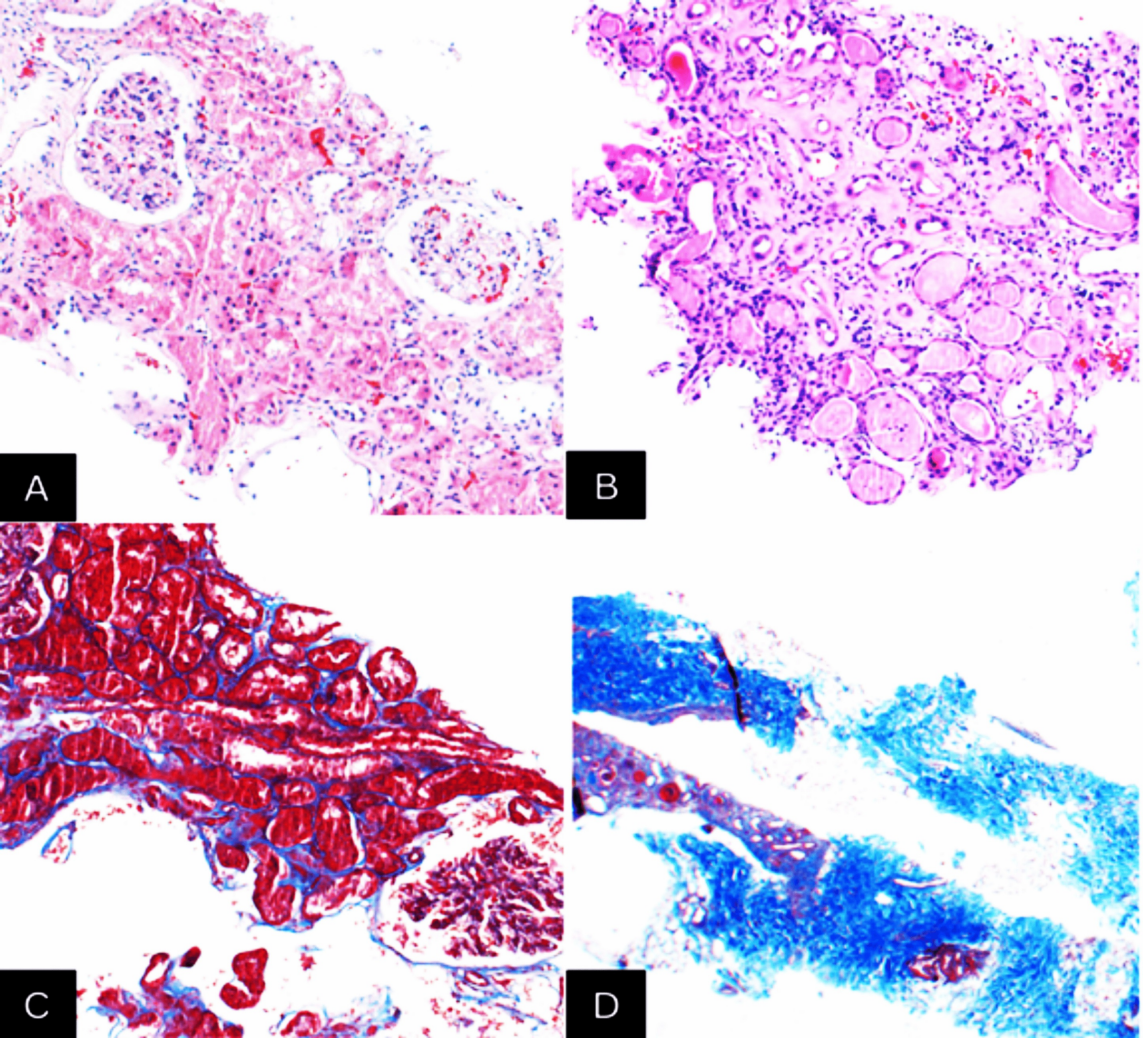
Renal histopathology showing (A) minimal interstitial inflammation (2017), (B) thyroidization pattern of tubular atrophy (2024), and (C) minimal interstitial fibrosis and tubular atrophy (2017). (D) Low-power image of trichrome staining highlighting severe interstitial fibrosis and tubular atrophy (2024)

There was concern of cardiorenal syndrome type 3, and the patient received intravenous (IV) diuretics with albumin infusions, with minimal improvement in volume status. Despite having two prior kidney biopsies which had identified IgA nephropathy, HIVICK, and primary MN, a third renal biopsy was strongly indicated due to concern of rapidly collapsing focal segmental glomerulosclerosis (FSGS), which is the most common form of HIV-associated glomerulonephritis (GN), and concomitant secondary MN from HIV and to determine the chronicity of disease for prognostication. The pathology showed PLA2R-positive membranous glomerulopathy with 70% severe interstitial fibrosis and tubular atrophy (IFTA). There was no evidence of HIVAN or HIVICK (Figure 2).

The patient remained significantly hypervolemic despite high doses of IV diuretics. His kidney function continued to deteriorate (Table 1), and due to the significant IFTA with a low probability of improvement of kidney function, the decision was made to initiate intermittent hemodialysis on day of admission (DOA) 8. However, this was deferred to DOA 12 due to sepsis secondary to *Klebsiella pneumoniae* bacteremia. Intermittent hemodialysis was started after the patient successfully cleared the bacteremia. Kidney function improved and remained stable (Table 1). The edema resolved, and the patient was discharged on DOA 15 with a creatinine of 3.9 mg/dL to continue with thrice-weekly outpatient hemodialysis.

## Discussion

The natural history of MN varies considerably. One-third of the patients with MN, who are usually categorized as low risk, will achieve spontaneous remission, one-third will develop non-progressive chronic kidney disease, and the remainder, typically classified as high risk, will progress to ESRD over 5-15 years if they received no treatment [7]. High-risk features predictive of progression include older age, elevated serum creatinine levels, and significant proteinuria exceeding 8 grams per liter.

As described in the case summary, our patient was diagnosed with primary MN seven years prior to this index admission. He met several high-risk criteria for progression to ESRD and was hence placed on immunosuppression. The patient achieved partial remission of proteinuria with rituximab after failed treatment with cyclosporine and prednisone. However, he was lost to follow-up for close to five years, during which time he was non-adherent to his medications. Upon evaluation during this index admission, the patient was hypervolemic and was managed for acute heart failure with echocardiographic findings of preserved ejection fraction. Kidney function and previous renal disease history made cardiorenal syndrome class 3, a diagnosis usually overlooked, more likely. A third biopsy was ordered to rule out secondary MN and rapidly collapsing FSGS and to provide prognostication. The pathology revealed severe (70%) IFTA, limiting immunosuppressive therapy as a treatment option. The Kidney Disease: Improving Global Outcomes (KDIGO) published guidelines in 2012 on the management of MN, recommending the initiation of immunosuppressive therapy for patients in the moderate- and high-risk categories to ensure remission and prevent progression to ESRD [8]. Persistent urinary protein excretion exceeding 4 g/day or remaining at or over 50% of the baseline value without progressive decline with antihypertensives or antiproteinuric therapy over six months or the presence of life-threatening symptoms or a rise in serum creatinine level by 30% or more over 6-12 months increases the patient's risk of progression to ESRD [8]. Other high-risk factors include advanced age, male sex, white race, a personal medical history of hypertension, and baseline elevated serum creatinine levels [9].

Immunosuppressive therapy in the progression of primary MN to ESRD has been extensively studied over three decades. In 1995, Ponticelli et al. followed patients with MN for 10 years after randomizing them to receive methylprednisolone and chlorambucil, finding sustained efficacy of this treatment regimen in improving renal outcomes long term [10]. In 2019, Fervenza et al. concluded that rituximab significantly increased remission rates and reduced proteinuria in patients with MN compared to standard therapy or supportive care [11]. Cattran et al., in a study published in 2017, also concluded that achieving complete remission (proteinuria <0.3 g/day) in MN correlates with improved long-term kidney function and reduced disease progression risk [12].

IFTA, i.e., greater than 50%, has been studied as a predictive factor for chronic renal failure [13]. However, it is not recommended to use IFTA as the sole prognosticating factor. Patients with advanced IFTA, lower serum creatinine, low proteinuria, and no other major risk factors for ESRD, including diabetes and hypertension, may still have a better renal prognosis and not progress to ESRD within five years [13]. However, in cases similar to our patient, who had IFTA of 70% associated with elevated serum creatinine, persistent significant proteinuria, non-adherence to medical therapy, and other major risk factors for ESRD such as hypertension and HIV, continuous immunosuppression has not been shown to be beneficial (Ho) [14].

## Conclusions

MN manifests with diverse clinical trajectories, influenced by individual risk factors such as age, baseline renal function, extent of proteinuria, immunosuppressant therapy, and adherence to medical therapy. While a significant proportion of patients may experience spontaneous remission or stability with non-progressive chronic kidney disease, others with risk factors such as advanced age, male gender, persistently low serum albumin, persistently elevated creatinine, significant proteinuria, and a history of pre-existing chronic kidney disease face a heightened risk of progressing to ESRD without intervention. Current guidelines advocate for the early initiation of immunosuppressive therapy in moderate- to high-risk cases to induce remission and mitigate renal deterioration. However, the efficacy of immunosuppression varies, particularly in cases with advanced IFTA, where treatment benefits may be limited and patients may require renal replacement therapy. This underscores the importance of personalized management strategies guided by comprehensive risk assessment and regular monitoring to optimize outcomes in patients with MN.
